# Rapid whole genome optical mapping of *Plasmodium falciparum*

**DOI:** 10.1186/1475-2875-10-252

**Published:** 2011-08-26

**Authors:** Matthew C Riley, Benjamin C Kirkup, Jake D Johnson, Emil P Lesho, Christian F Ockenhouse

**Affiliations:** 1 Walter Reed Army Institute of Research, Division of Malaria Vaccine Development, Silver Spring, Maryland, USA; 2 Walter Reed Army Institute of Research, Division of Bacterial and Rickettsial Diseases, Silver Spring, Maryland, USA; 3 Walter Reed Army Institute of Research, Division of Experimental Therapeutics, Silver Spring, Maryland, USA; 4 Uniformed Services University of the Health Sciences, Silver Spring, Maryland, USA

## Abstract

**Background:**

Immune evasion and drug resistance in malaria have been linked to chromosomal recombination and gene copy number variation (CNV). These events are ideally studied using comparative genomic analyses; however in malaria these analyses are not as common or thorough as in other infectious diseases, partly due to the difficulty in sequencing and assembling complete genome drafts. Recently, whole genome optical mapping has gained wide use in support of genomic sequence assembly and comparison. Here, a rapid technique for producing whole genome optical maps of *Plasmodium falciparum *is described and the results of mapping four genomes are presented.

**Methods:**

Four laboratory strains of *P. falciparum *were analysed using the Argus™ optical mapping system to produce ordered restriction fragment maps of all 14 chromosomes in each genome. *Plasmodium falciparum *DNA was isolated directly from blood culture, visualized using the Argus™ system and assembled in a manner analogous to next generation sequence assembly into maps (AssemblyViewer™, OpGen Inc.^®^). Full coverage maps were generated for *P. falciparum *strains 3D7, FVO, D6 and C235. A reference *P. falciparum in silico *map was created by the digestion of the genomic sequence of *P. falciparum *with the restriction enzyme AflII, for comparisons to genomic optical maps. Maps were then compared using the MapSolver™ software.

**Results:**

Genomic variation was observed among the mapped strains, as well as between the map of the reference strain and the map derived from the putative sequence of that same strain. Duplications, deletions, insertions, inversions and misassemblies of sizes ranging from 3,500 base pairs up to 78,000 base pairs were observed. Many genomic events occurred in areas of known repetitive sequence or high copy number genes, including *var *gene clusters and *rifin *complexes.

**Conclusions:**

This technique for optical mapping of multiple malaria genomes allows for whole genome comparison of multiple strains and can assist in identifying genetic variation and sequence contig assembly. New protocols and technology allowed us to produce high quality contigs spanning four *P. falciparum *genomes in six weeks for less than $1,000.00 per genome. This relatively low cost and quick turnaround makes the technique valuable compared to other genomic sequencing technologies for studying genetic variation in malaria.

## Background

Malaria is caused by various species of the genus *Plasmodium*, the most prevalent and deadly of which is *Plasmodium falciparum*[1,2]. With ~40% of the world's population at risk for malaria, efforts in prevention, eradication and treatment of the disease are globally vital [2,3]. Despite efforts to develop vaccines and drugs to combat malaria, vaccine escape and drug resistance continue to be a problem [1,4,5]. The ability to compare whole genomes could assist in these efforts, as genetic variation and recombination have been shown to facilitate antigen diversity, immune escape and evolution of anti-malarial drug resistance [4,6-9]. Proposed mechanisms for these events include chromosome translocation and recombination, segmental duplication and CNV [10-13]. Ideally, fully sequenced and assembled genomes would be a way to study these mechanisms; however, this continues to be expensive and labour intensive [12-14]. Optical mapping provides an alternative to study these events, but to date has not been used for this purpose in malaria, partly due to the impracticality of scaling up previous mapping techniques to multiple genomes [15].

**Figure 1:**
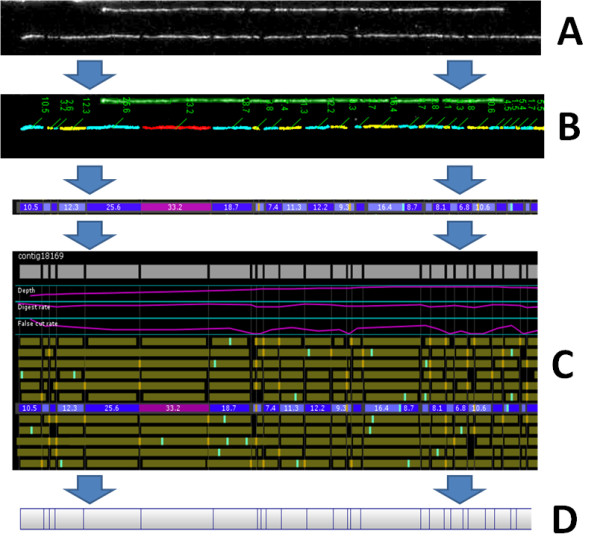
**Generating an Optical Map**; After DNA is bound and digested, imaging of molecules is performed (A). The fragments are assigned sizes (B) and assembled (C) to generate a consensus optical map (D). Imaging, cut site and DNA length prediction is automatic, and represented here as spaces between the colored, labeled fragments.

Optical mapping is a technique during which visualization of high molecular weight DNA cut by restriction enzymes is used to create a scaffold fragment pattern across a genome (Figure 1). The first and most critical step in this process is the purification of extremely high molecular weight DNA (150Kb-1.6Mb). This was accomplished in this case by using magnetic bead technology. The DNA is then bound to a glass surface and subjected to a pre-determined restriction enzyme and labelled using a fluorescent dye. These digested, bound molecules are then imaged and relative sizes are assigned to generate a cut site pattern. These patterns are used to assemble the molecules against each other, generating a digital representation of fragment pattern alignments, or an "optical map"[16].

The 14 chromosome, 14 Mbp *P. falciparum *strain 3D7 genome was published in 2002, and to date this is the only complete, assembled genome sequence for this organism [14]. Optical mapping of DNA was used to assist in assembly of this published genome sequence, but until now has not been widely used on any other species or strains [15,17]. The Argus™ optical mapping system represents a radical advancement in whole genome optical mapping technology, and is being widely used for studies in bacterial identification, evolution, comparative genomics and genome assembly [18-22]. By developing new parasite isolation techniques and DNA extraction protocols, stated Argus™ limitations were surpassed to produce whole genome maps of malaria genomes for under $1,000.00 each, fully assembled in less than one week.

## Results

### Parasite isolation and DNA extraction

**Figure 2:**
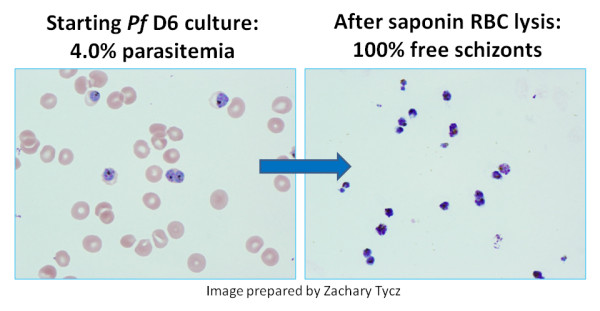
**Purification of Malaria Parasites**; A: *Plasmodium falciparum *D6 culture at 4.0% parasitaemia. Notice free red blood cells (RBCs) schizonts inside RBCs. B: 100% free schizonts after saponin RBC lysis. Notice faint ghost (lysed) RBCs.

Two vaccine research *P. falciparum *strains and two drug resistance research strains were selected for this work. Strains 3D7 and FVO are commonly used in vaccine development and 3D7 allowed the direct comparison of its optical map and the sequence based in silico map which was used as a reference [14]. Strains D6 (NC_004317) and C235 were selected based on their opposing anti-malarial drug resistance profiles; D6 is generally considered drug sensitive with minor mefloquine resistance, while C235 is considered a multi-drug resistant strain [Unpublished Data]. All four parasites are culture-adapted strains and were grown to 4-6% parasitaemia in 6 mL standard blood culture prior to purification. Isolation of late ring- early schizont-stage parasites was performed by multiple 4°C wash/centrifuge cycles after initial lysis with 0.1% saponin in PBS. The blood culture was centrifuged at 2,486 rpm (860 rcf) and 4°C for 6 minutes and the pellet resuspended in one-half starting volume of ice cold 0.1% saponin/PBS (w/v). This solution was allowed to incubate on ice for 10 minutes and then centrifuged again at 2,486 rpm (860 rcf) and 4°C for 6 minutes. After removal of supernatant, the pellet was resuspended in ice cold DNAse free water and centrifuged again at 2,486 rpm (860 rcf) and 4°C for 6 minutes. This protocol resulted in release of malaria parasites and elimination of RBCs (Figure 2). This pellet was resuspended in 50 μL of ice cold DNAse free water and used directly in DNA extraction.

DNA extraction was performed on this purified parasite sample according to the Gram-negative Bacterial DNA Preparation protocol available from Opgen, Inc.^®^. The parasites were lysed and resulting DNA was mixed with magnetic beads, subjected to a variety of wash buffers and eluted off the beads using wide bore pipette tips. Modifications to the manufacturer's protocol included addition of a 20 minute extension of initial lysis time, reduction in elution volume from 90 μL to 75 μL, and an extra two elutions at 65°C for 15 minutes each. Between 4 μL and 6 μL DNA was applied to mapcards (cards run on the Argus™) and run on the Argus™ system per manufacturer's specifications, after proper quality controls checks and appropriate dilutions using Opgen^® ^QCards.

### Mapset filter and contig assembly

**Figure 3:**
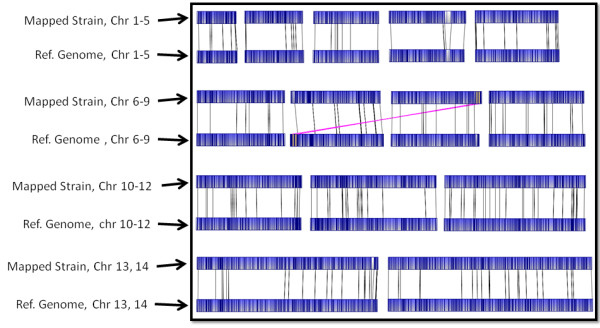
**Whole Genome Optical Map Alignment**; All 14 chromosomes of the *in silico *3D7 reference genome optical map (bottom aligned maps, A) align to the 3D7 *in vivo *optical map (top maps, B). A large expansion on chromosome 4, a expansion at a gap site on chromosome 13 and a misassembly between chromosomes 7 and 8 are observed.

Contig assembly was performed using the Argus™ software, but with changes to default settings. The mapset (total dataset generated from a single run) was filtered for minimum molecule (chromosome segment imaged) size (> 150 Kb), minimum fragments per molecule (>12) and minimum molecule quality (>0.4). Each genome was able to be assembled on as few as two mapcards; however, in some cases more cards were used in an attempt to resolve difficult telomeric chromosome ends. The data from each additional run on the same strain were combined together for final assembly. Assembly was conducted using either the "fastest" setting or "slower" setting, with removal of default circularization parameters. Partial assembly results were saved when 14 contigs became apparent by having >50 molecules each. Contigs were split off and reassembled against the original mapset individually using the "Find Hits" feature. Contigs were considered "finished" when no additional molecules were added by subsequent reassemblies. The only areas that did not pass default Argus™ quality control (QC) settings were telomeric repeats near some chromosome ends. Chromosome ends that were not blunt were visually inspected and any questionable molecule was removed from the final map. Finished contigs were imported and aligned using Opgen^® ^MapSolver™ software with default alignment settings (Figure 3).

### Genome alignment and comparative genomics

**Figure 4:**
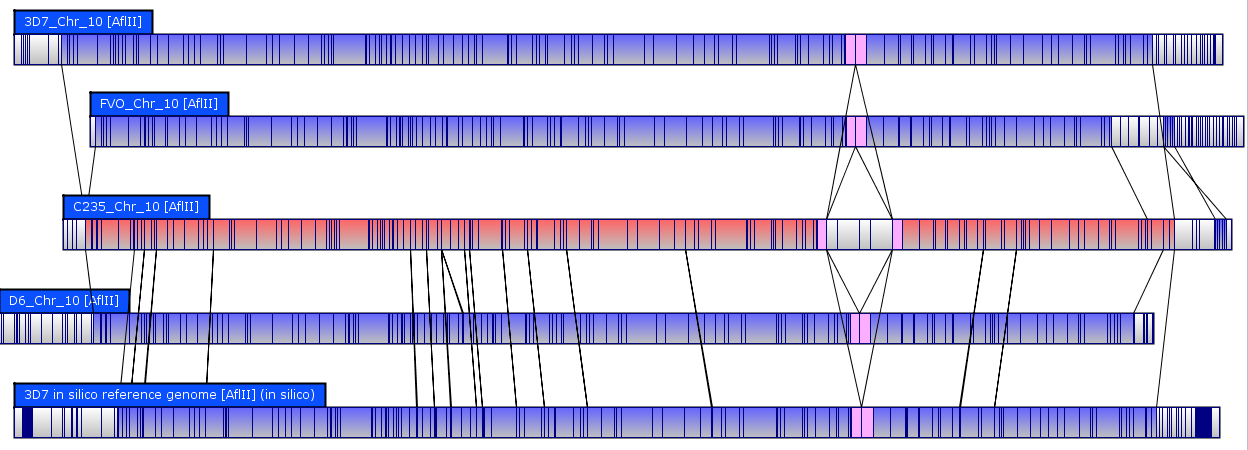
**Unique DNA Insertion in Chromosome 10 of Strain C235**; Strain C235, generally considered to be multidrug resistant, displays a single large unique insertion when compared to the other strains mapped and the *in silico *reference map. Areas of matching alignment are highlighted in blue, whereas areas in white indicate no match to the other maps. The insertion seems to represent a single sequence of about 35Kb that was duplicated back to back.

Each genome was aligned against the 3D7 reference genome and genomic differences were noted and compared against the other strains (Table 1). Relative to the 3D7 reference DNA sequence, the 3D7 optical map showed two probable misassemblies, one gene duplication and two insertion/deletion events. Overall, this accounts for ~304 Kb of unaligned sequence. The multi-drug resistant strain C235 shows a ~70 Kb insertion that is of particular interest as it is the largest observed difference and is completely unique to this genome (Figure 4).

**Table 1:** Summary of Results

Genome	Total Length (Mb)	Geographic Area	Variations to Reference^a^	Maximum Single Variation^b^
3D7 Reference	23.264	West Africa	N/A	N/A

3D7 Optical Map	23.165	West Africa	4	46.0Kb

D6	21.958	East Africa	5	43.6Kb

FVO	22.158	Southeast Asia	4	41.4Kb

C235	21.734	Southeast Asia	5	77.5Kb

The total megabases (Mb) of each genome predicted from alignment against the in silico optical map (or reference), the geographic area each strain was isolated from, the number of variations from each map to the reference genome and the largest (in Kb) observed difference. Variation under 2Kb and telomeric DNA were not included.

^a^represents independent differences observed against the reference genome using default assembly parameters. Results can vary by settings.

^b^the maximum number of kilobases for a single genomic event outlined in the study as compared to the *in silico *reference genome.

**Figure 5:**
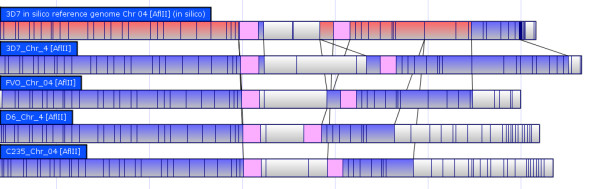
**Chromosome 4 Region of Variation in all Strains**; Compared to the reference genome, all strains mapped generally align (in blue) to the reference genome (red). However, the white area indicates no alignment in any genome to another, indicating a high degree of variation.

Each genomic event was aligned to the reference sequence from PlasmoDB [23], and the DNA sequence for the insertion (or flanking DNA for deletions) was referenced to associated genes. In many cases *var *or *rifin *complexes were associated with these events, in particular PfEMP1, a known source of antigenic variation [24]. One identified *var *cluster of known variation on chromosome 4 is shown in Figure 5 in the four genomes mapped aligned to the reference genome.

## Discussion

The major hurdle to optically mapping *P. falciparum *was sample preparation. Because the parasites can be grown only in blood culture, there is a possibility of contamination of the sample by human white blood cells or nucleated erythrocytes. As there is no amplification involved in this technique, even a single human cell (3,300 Mb DNA/cell) could overwhelm the sample with human DNA and mask the comparatively small 24 Mb/cell malaria genome. This was overcome using the parasite isolation technique described in the methods. The success of this method was such that no contigs in the assemblies failed to match a region of the sequenced *Plasmodium *genome.

**Figure 6:**
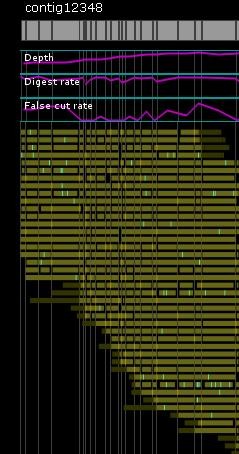
**Optical Resolution of Blunt Chromosome Ends**; Unlike bacterial optical maps, malaria DNA is not circular. Telomeric repeats near the ends of some chromosomes can cause the map to be truncated, unless a "blunt" end is observed as shown in this figure. "Blunt" ends in optical mapping refer not to enzyme cut sites but the true end of a chromosome as depicted by having all individual molecules "end" at the same sequence.

Some chromosome ends did not readily align to the reference strain. These unaligned map ends often had poor QC scores and low coverage. Using AflII as a mapping enzyme would result in multiple small AflII fragments at the end containing telomeric repeats which would be not resolved. Thus, while there may be more DNA at the ends of some chromosomes than is represented in our optical maps, the DNA and related cut site patterns internal to these ends are correct. Blunt ends were observed at some contig ends and passed QC indicating that we resolved the full length of the chromosome (Figure 6).

**Figure 7:**
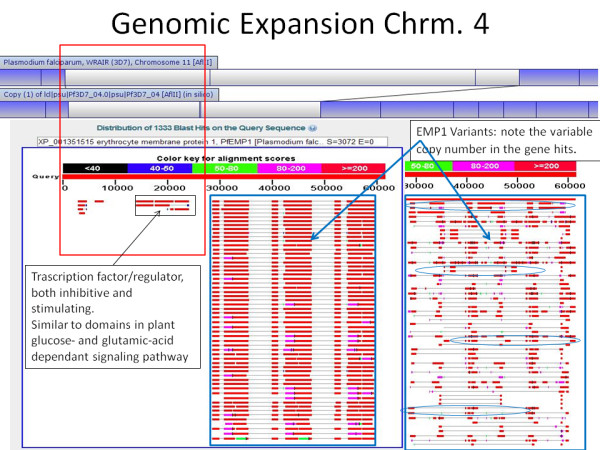
**Chromosome 4 Variable Region Associated with *var *Cluster**; The variable region on chromosome 4 shown in Figure 6 contains DNA that matches to an area of known variation. Here, you can see the BLAST result of the repetitive sequence found in this region. [http://blast.ncbi.nlm.nih.gov/Blast.cgi]

**Figure 8:**
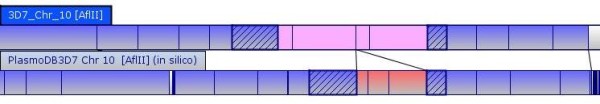
**Chromosome 10 Duplication of PfEMP1**; The segmental duplication observed in Figure 3 is located on chromosome 10 and is potentially an exact duplication of the gene *PfEMP1*.

Complete chromosome end translocations were observed in C235 and FVO, indicating this could be a frequently observed difference between distantly related strains. A single region on chromosome 4 varied significantly in length (up to 65Kb) among the strains studied. It includes a known *var *gene cluster in the reference genome (Figure 7) [6]. The *var *clusters (which contain variants of PfEMP1) are known to be associated with antigenic variation and are thought to be a candidate for vaccine development [6,9,24]. In the 3D7 optical map, an exact duplication of PfEMP1 was observed in comparison to other strains and the reference genome (Figure 8). PfEMP1 is a known source of antigenic variation, and while segmental duplication has been implicated as a possible mechanism, until now it has not been directly observed [24]. Strain C235 is considered multi-drug resistant and thus any unique genomic events compared to susceptible strains are of particular interest [Unpublished Data]. While there were several non-unique insertions and deletions compared to the reference genome, we did observe a single 70Kb insertion on C235 chromosome 10 that does not have a similar pattern in any of the other genomes mapped (Figure 4). This insert seems to be a 35Kb segment of DNA that was duplicated back to back, which is potentially meaningful as drug resistance is often associated with gene duplication and CNV [4]. Using Mapsolver™ UPGMA phylogenetic trees for individual chromosomes, 3D7 was found to be most similar to D6 (9/14 chromosomes), while C235 and FVO were most closely related (9/14 chromosomes). These relationships most likely reflect the geographic regions that each parasite is from (Table 1).

## Conclusions

Optical genomic mapping technology represents a significant advancement in comparative genomic research of malaria parasites. The optical maps of the four *P. falciparum *strains presented here demonstrate significant genome variation, much of which can be traced to regions known to contain coding sequence implicated in antigenic variation. This confirms findings generated via more difficult and expensive research methods that have suggested chromosomal translocations and segmental duplications are associated with immune escape and drug resistance in malaria [6-8,10,11]. Optical mapping resolves these types of genomic events and the techniques described in this paper allow for inexpensive, rapid, robust production of whole genome optical maps for malaria. This method also opens the door for assistance with genome assembly, as has been demonstrated with optical mapping of other large genomes [25-27]. The development of optical maps and fully assembled malaria genomes will increase knowledge of genetic variation in malaria parasites and thus enhance the ability to combat this disease.

## Competing interests

The authors declare that they have no competing interests.

## Authors' contributions

MCR performed parasite isolation and DNA purification, Optical mapping, data analysis and drafted the manuscript. BCK helped conceive the study, participated in its design and coordination and helped draft the manuscript. JJ assisted with parasite isolation, provided strains C235 and D6, characterized their drug resistance and helped draft the manuscript. EL participated in the design and coordination of the study and provided training and support for the optical mapping. CO helped conceive of the study, directed the study design and provided strains 3D7 and FVO. All authors read and approved the final manuscript.
